# Intraoperative oxygenation in adult patients undergoing surgery (iOPS): a retrospective observational study across 29 UK hospitals

**DOI:** 10.1186/s13741-018-0098-3

**Published:** 2018-07-24

**Authors:** Clare M. Morkane, Helen McKenna, Andrew F. Cumpstey, Alex H. Oldman, Michael P. W. Grocott, Daniel S. Martin, Pan London Perioperative Audit and Research Network (PLAN), Louise Carter, Cyrus Razavi, Ryan Howle, Alex Eeles, Kate C. Tatham, Victoria Winter, Lena Al-Shammari, Leda Lignos, Gagandeep Dhotar, Emma Karsten, Justine Lowe, Noel Young, Lindsey Iles, Colin Coulter, Michael Shaw, Liam Gleeson, Liana Zucco, Charlie Cox, Amanda Bruce, John N. Cronin, James Arlidge, Rachel Krol, Rasha Abouelmagd, Phil Dart, Mohamed Ahmed, Kathy Shammas, Carly Webb, Luke Foster, Rafi Kanji, Darragh Hodnett, Lusha Suntharanathan, Amy Sangam, Zain Malik, Eleanor Jeffreys, Jonathan Williamson, Marika Chandler, Nick Dennison, Jan Schumacher, Kariem El-Boghdadly, Peter Odor, Helen Laycock, Sibtain Anwar, Harriet Wordsworth, Alex Wickham, Shaima Elnour, Edward Burdett, Sioned Phillips, Matt Oliver, Carolyn Johnston, Mitul Patel, Kate Grailey, Queenie Lo, Benjamin Frost, James O’Carroll, Hew D. Torrance, Vimal Grover, Chris Whiten, Justine Lowe, Matthew C. Dickinson, Vanessa Cowie, Richard George, Julian Giles, Otto Mohr, Ahmer Mosharaf, Jon Brammall

**Affiliations:** 1 Division of Surgery and Interventional Science (University College London) and Royal Free Perioperative Research Group, Department of Anaesthesia, Royal Free Hospital, 3rd Floor, Pond Street, London, NW3 2QG UK; 2 University of Southampton/University Hospital Southampton and NIHR Biomedical Research Centre, Tremona Rd, Southampton, SO16 6YD UK; 3 University Hospital Southampton, Tremona Rd, Southampton, SO16 6YD UK

**Keywords:** Hyperoxia, Oxygen, Surgical procedures, Operative

## Abstract

**Background:**

Considerable controversy remains about how much oxygen patients should receive during surgery. The 2016 World Health Organization (WHO) guidelines recommend that intubated patients receive a fractional inspired oxygen concentration (FIO_2_) of 0.8 throughout abdominal surgery to reduce the risk of surgical site infection. However, this recommendation has been widely criticised by anaesthetists and evidence from other clinical contexts has suggested that giving a high concentration of oxygen might worsen patient outcomes. This retrospective multi-centre observational study aimed to ascertain intraoperative oxygen administration practice by anaesthetists across parts of the UK.

**Methods:**

Patients undergoing general anaesthesia with an arterial catheter in situ across hospitals affiliated with two anaesthetic trainee audit networks (PLAN, SPARC) were eligible for inclusion unless undergoing cardiopulmonary bypass. Demographic and intraoperative oxygenation data, haemoglobin saturation and positive end-expiratory pressure were retrieved from anaesthetic charts and arterial blood gases (ABGs) over five consecutive weekdays in April and May 2017.

**Results:**

Three hundred seventy-eight patients from 29 hospitals were included. Median age was 66 years, 205 (54.2%) were male and median ASA grade was 3. One hundred eight (28.6%) were emergency cases. An anticipated difficult airway or raised BMI was documented preoperatively in 31 (8.2%) and 45 (11.9%) respectively. Respiratory or cardiac comorbidity was documented in 103 (27%) and 83 (22%) respectively. SpO_2_ < 96% was documented in 83 (22%) patients, with 7 (1.9%) patients desaturating < 88% at any point intraoperatively. The intraoperative FIO_2_ ranged from 0.25 to 1.0, and median PaO_2_/FIO_2_ ratios for the first four arterial blood gases taken in each case were 24.6/0.5, 23.4/0.49, 25.7/0.46 and 25.4/0.47 respectively.

**Conclusions:**

Intraoperative oxygenation currently varies widely. An intraoperative FIO_2_ of 0.5 currently represents standard intraoperative practice in the UK, with surgical patients often experiencing moderate levels of hyperoxaemia. This differs from both WHO’s recommendation of using an FIO_2_ of 0.8 intraoperatively, and also, the value most previous interventional oxygen therapy trials have used to represent standard care (typically FIO_2_ = 0.3). These findings should be used to aid the design of future intraoperative oxygen studies.

## Background

Approximately 3 million patients undergo general anaesthesia in the UK each year and are routinely given supplemental oxygen as part of this procedure (Sury et al. 2014). This makes oxygen one of the most commonly used drugs during surgery, yet there still remains considerable uncertainty about how much oxygen patients should receive during the perioperative period. In November 2016, the World Health Organization (WHO) recommended that all intubated patients receive a fractional inspired oxygen concentration (FIO_2_) of 0.8 throughout surgery and for up to 6 h in recovery, as this might reduce patients’ risk of developing a surgical site infection (SSI) later (Allegranzi et al. 2016). However, this recommendation has already been widely criticised by anaesthetists (Ball et al. 2017; Myles and Kurz 2017), and a Cochrane systematic review and meta-analysis of 28 trials published in 2015 concluded that robust evidence is lacking for a beneficial effect of using a high FIO_2_ to reduce SSIs (Wetterslev et al. 2015). In fact, this meta-analysis suggested using a high FIO_2_ during surgery could increase the risk of adverse events, including mortality, after long-term findings of the PROXI study (the largest study included in this review) reported significantly higher 2-year mortality rate in patients with abdominal malignancy who received an FIO_2_ of 0.8 (Wetterslev et al. 2015; Meyhoff et al. 2012).

WHO’s recommendations would also appear to contradict the consensus opinion in other clinical contexts; concerns have been raised about potential harms associated with hyperoxaemia (defined by others as an arterial oxygen partial pressure (PaO_2_) > 13.3 kPa or 100 mmHg (Damiani et al. 2014a)) after myocardial infarction, after cardiac arrest and also in critical illness (Farquhar et al. 2009; Damiani et al. Dell’Anna et al. 2014). Within 15–30 min of onset, FiO_2_ 0.8–1.0 has also been demonstrated to induce atelectasis (Edmark et al. 2003), systemic vasoconstriction (Reinhart et al. 1991), coronary vasoconstriction (McNulty et al. 2005) and (within hours) pulmonary inflammation as well (Davis et al. 1983). Furthermore, chemical free radicals generated from oxygen (known as reactive oxygen species, ROS) can also avidly oxidise proteins, lipids or DNA resulting in cellular oxidative stress—an integral part of the normal surgical stress response that may also be associated with the development of multiple post-operative complications (Kücükakin et al. 2009). A large meta-analysis of over 16,000 patients concluded that high-quality evidence now shows liberal oxygen therapy in acutely ill adults increases mortality without improving other patient-important outcomes, suggesting that supplemental oxygen administration might become unfavourable above an SpO_2_ range of 94–98% (Chu et al. 2018).

Currently, there is limited data describing the intraoperative oxygen administration practices of UK anaesthetists. Given the current controversy surrounding WHO’s recommendations for perioperative oxygen use, the aim of this multi-centre observational study was to characterise practice as regards the administration of oxygen to patients undergoing major surgery and to describe intraoperative arterial oxygenation during general anaesthesia.

## Methods

A multi-centre retrospective observational study was conducted across 29 hospitals in London and parts of the South Coast of England affiliated with two trainee-led research networks: PLAN (Pan-London Perioperative Audit and Research Network—http://www.uk-plan.net) or SPARC (South Coast Perioperative Audit and Research Collaborative—http://wessex-sparc.com). The project was confirmed to be a clinical service evaluation by the Royal Free London and Southampton NHS Trust Clinical Governance departments, and research ethics committee approval and individual patient consent were not required. Appropriate approval was secured from the clinical governance department in each participating hospital.

Patients aged 18 years and over undergoing general anaesthesia for elective or emergency operations necessitating the insertion of an arterial cannula and subsequent arterial blood gas (ABG) monitoring were included. Patients receiving cardiopulmonary bypass were excluded (as during bypass, oxygen administration is often not controlled by the anaesthetist).

Data collection took place over five consecutive weekdays in April and May 2017; flexibility in the data collection window ensured maximum compliance locally. Anaesthetic trainees not involved in the clinical care of the patients collected data from the anaesthetic record. Patients were identified and assessed for inclusion daily from departmental operating lists. A retrospective review of anaesthetic charts and arterial blood gases was performed after the patients in question had been moved from theatre to the recovery area, ward or intensive care unit. Oxygenation data (including intraoperative SpO_2_, FIO_2_ and PaO_2_ values), together with intraoperative positive end-expiratory pressure (PEEP) values and patient demographics, were collected using paper case report forms, held securely and treated as strictly confidential according to NHS policies.

Statistics were calculated using IBM SPSS Statistics, Version 24.0, Armonk, NY: IBM Corp. Data were examined for normality using the Shapiro-Wilk test. Unpaired data were compared using the Wilcoxon-Mann-Whitney *U* test and Kruskal-Wallis tests. Correlation was tested with Spearman’s rank correlation coefficient. All tests were two-tailed, and significance was taken as *p* < 0.05. Continuous data were presented as median (IQR) and categorical data as number (percentage). Cumulative oxygen dose was determined in patients for whom more than one ABG was recorded, by calculating the area under the curve between the times of the first and final ABGs, in a plot of recorded PaO_2_ as a function of time, with T0 equal to the time of the initial ABG.

## Results

Data from 378 anaesthetic cases were contributed from 29 hospitals across London and Wessex. Results were reported from 17 (58.6%) district general hospitals (DGHs), 8 (27.6%) teaching hospitals and 4 (13.8%) speciality hospitals. Paper-based anaesthetic records were used in 334 (88.4%) cases, with electronic records contributed by three sites. The median patient age was 66 years (IQR 52–74). Patient demographics and clinical details along with operation characteristics are shown in Table 1.

**Table 1:** Patient and operation characteristics

Variable		Patient/surgical characteristics number (%)
Gender	Male	205 (54.2%)
	Female	173 (45.8%)
ASA classification	1	17 (4.5%)
	2	158 (41.8%)
	3	153 (40.5%)
	4	34 (8.99%)
	5	5 (1.3%)
	Not recorded	11 (2.9%)
Documented respiratory disease	Asthma	37 (9.8%)
	COPD	31 (8.2%)
	Obstructive sleep apnoea	10 (2.6%)
	Other	25 (6.6%)
Documented cardiovascular disease	Hypertension	145 (38.4%)
	Ischaemic heart disease	49 (13%)
	Atrial fibrillation	31 (8.2%)
	Congestive cardiac failure	16 (4.2%)
	Valvular disease	18 (4.8%)
	Other	34 (9%)
National Confidential Enquiry into Patient Outcome and Death (NCEPOD) classification	Elective	270 (71.4%)
	Urgent/immediate/expedited	108 (28.6%)
Surgical specialty	Upper gastrointestinal/colorectal/general/breast	111 (29.4%)
	Urology/renal (including renal transplantation)	34 (9%)
	Vascular	37 (9.8%)
	Orthopaedics/spinal	29 (7.7%)
	Hepatopancreaticobiliary/liver transplant (including liver transplantation)	32 (8.5%)
	Ear nose and throat/maxillofacial	17 (4.5%)
	Neurosurgery	45 (11.9%)
	Gynaecology	23 (6.1%)
	Cardiothoracic	23 (6.1%)
	Plastics	2 (0.5%)
	Other	25 (6.6%)

Surgical duration ranged from 1 to 13 h with a median of 4 h. Estimated blood loss was > 1000 ml in 31 (8.2%) patients. In total, 824 arterial blood gases were analysed. The number of ABGs recorded for each patient ranged from 1 to 13, with a median of 2. SpO_2_ of < 96% was documented in 83 (22%) patients, with only 7 (1.9%) patients desaturating to < 88% at any point during the operation. Table 2 illustrates values for SpO_2_, PaO_2_ and haemoglobin concentration in the first five ABGs for each patient.

**Table 2:** Oxygenation and haemoglobin values from the first five sequential arterial blood gas samples

ABG number	Median FIO_2_ (IQR)	Median PaO_2_ in kPa (IQR)	Median P:F ratio in kPa (IQR)	Median haemoglobin concentration g/l (IQR)
1 (*n* = 378)	0.5 (0.45–0.59)	24.5 (16.7–32.6)	51.2 (36.9–66.0)	113 (100–126)
2 (*n* = 227)	0.49 (0.4–0.54)	23.4 (17.4–29.5)	50.6 (38.1–62.9)	111 (100–124)
3 (*n* = 116)	0.46 (0.4–0.51)	25.7 (19.6–29.2)	54.5 (43.3–65.2)	108 (99–119)
4 (*n* = 51)	0.47 (0.4–0.5)	25.4 (20.7–30.8)	57.2 (43.9–71.1)	111 (93–153)
5 (*n* = 24)	0.49 (0.3–0.51)	26.3 (23.4–29.3)	58.6 (45.3–66.7)	99 (93–116)

**Fig. 1:**
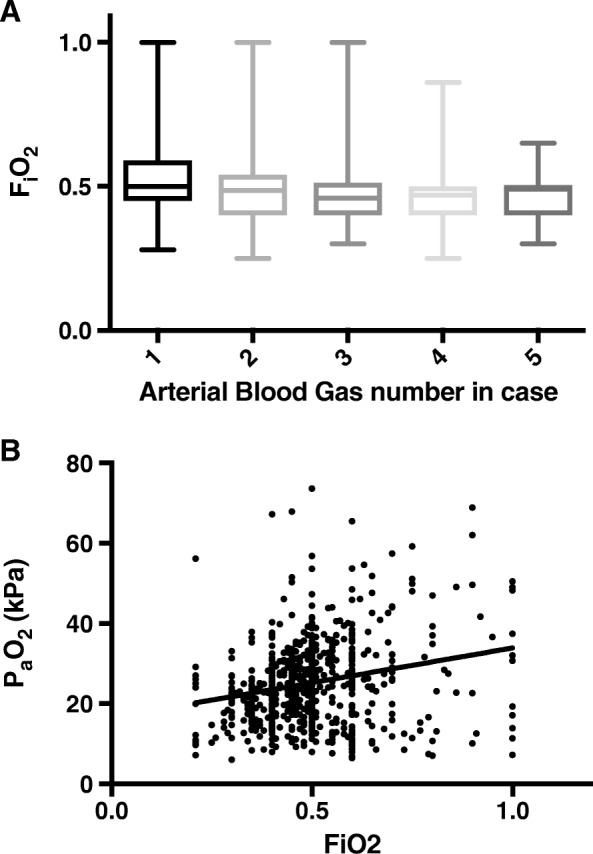
Intraoperative oxygenation illustrated by **a** box and whisker plot illustrating FIO_2_ administered over first five ABGs. Boxes are drawn between 25th and 75th percentiles with the median represented by a line and the whiskers indicating the minimum and maximum values. **b** Scatter plot and linear relationship between FIO_2_ and PaO_2_ for each ABG. The continuous line represents the relationship between partial pressure of arterial oxygen recorded and the fraction of inspired oxygen delivered (*r* = 0.22, *p* ≤  0.001)

The median PaO_2_ and FIO_2_ for all analysed ABGs combined were 24.7 kPa (IQR 17.9–30.8) and 0.50 kPa (IQR 0.41–0.55) respectively. There was no significant difference in the PaO_2_ recorded across sequential ABGs (*p* = 0.23). Median FIO_2_ was also consistent across sequential ABGs, although the variation about the median decreased by the fifth ABG (Fig. 1). Figure 1b demonstrates marked spread of data and a weak positive association between measured PaO_2_ and FIO_2_ (*r* = 0.22, *p* ≤ 0.001). Supraphysiological values for PaO_2_ (defined as > 13.3 kPa) were observed in 734 (89%) ABGs. Of the 769 ABGs for which the corresponding FIO_2_ was recorded, an FIO_2_ ≥ 0.8 was administered on 32 (4.2%) occasions. Of these 32 occasions, 20 (62.5%) were at the time of taking the baseline arterial gas, closest to induction of anaesthesia.

**Fig. 2:**
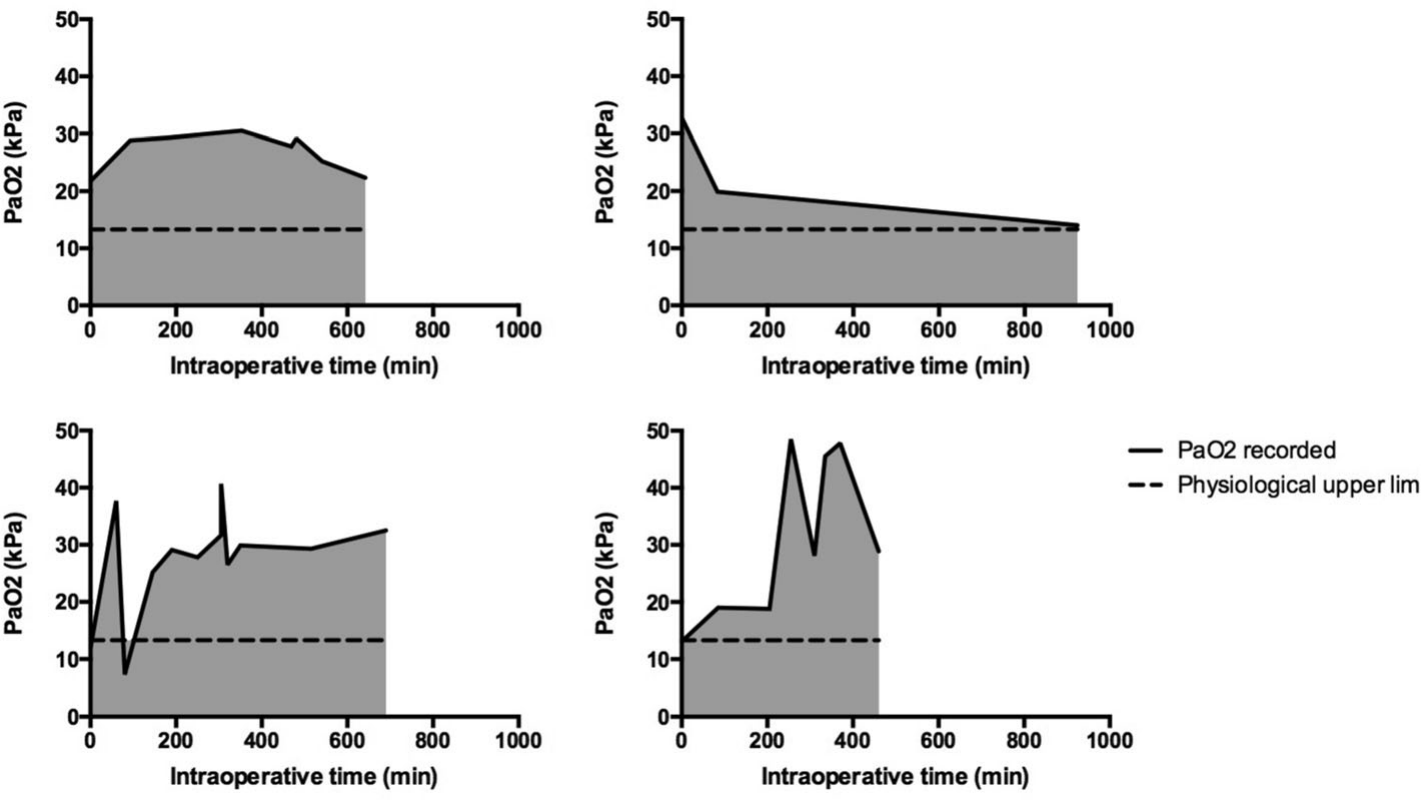
Sample traces demonstrating of cumulative oxygen dose for four individual patients. The solid line represents the actual PaO_2_ recorded in successive blood gases, whilst the dashed line represents the physiological upper limit (13.3 kPa). Area under the curve (shaded) was calculated between the times of the first and final ABGs

The median cumulative oxygen dose, calculated for those patients for whom at least two ABGs were documented (*n* = 223), was 3824 kPa min (IQR 2121–6923) over a median time of 159 min (IQR 91–291). The administration of 13.3 kPa O_2_ over the same time period would have resulted in a median cumulative oxygen dose of 2088 kPa. Representative traces of the cumulative oxygen dose administered to four individual patients are illustrated in Fig. 2.

Positive end-expiratory pressure (PEEP) was recorded in 287 (75.9%) of cases, and the median PEEP administered was 5 cmH_2_O (range 0–12). A PEEP of 4, 5 or 6 cmH_2_O was administered in 207 (72.1%) of cases where PEEP was recorded. A change in the level of PEEP administered was documented during only 28 cases.

## Discussion

These results demonstrate that the amount of oxygen anaesthetists administer to adult patients undergoing major surgery in the UK currently varies widely—the recorded FIO_2_ ranged from 0.25 to 1.0 throughout surgery. In many patients, FIO_2_ was nearer 0.5 for the duration of surgery, resulting in PaO_2_ values of approximately 25 kPa throughout.

An FIO_2_ of 0.5 is much higher than “standard” therapy used for control groups (where FIO_2_ is typically 0.3) in previous studies of “high” versus “standard” oxygen therapy (Wetterslev et al. 2015) and also considerably less than the WHO now recommends (Allegranzi et al. 2016). Interestingly, the findings from this UK-based sample exactly match values recently reported as representing current practice in the Cleveland Clinic, USA (Kurz et al. 2018). These results are also similar to the LAS VEGAS study (a prospective cross-sectional study of 9808 patients from 29 different countries) where half of all patients received an FIO_2_ between 0.4 and 0.6 and one third between 0.6 and 0.8 Rogerson et al. (2017). LAS VEGAS also reported a median PEEP of 5 cmH_2_O (the value in > 50% cases where PEEP was recorded in this study) suggesting this also represents the current “standard” of practice Rogerson et al. (2017).

In many other clinical contexts, including on the critical care unit, PaO_2_ values around 25 kPa would likely be classed as moderate hyperoxia rather than normoxia (Damiani et al. 2014a). However, median intraoperative PaO_2_ values of approximately 25 kPa are consistent with an earlier single UK centre pilot study carried out by our group, reporting a mean PaO_2_ of 24.4 kPa in 75 surgical patients over a 6-week period (Martin and Grocott 2015), and observational data also suggests that current practice still favours hyperoxaemia in critically ill patients (de Jonge et al. 2008; Eastwood et al. 2012).

Intraoperative hyperoxaemia may be a consequence of several factors. Firstly, up to now, evidence associating hyperoxia under anaesthesia with harm has been relatively limited. However, high intraoperative FIO_2_ has been retrospectively associated in a dose-dependent manner with increased post-operative respiratory complications and with increased mortality (Staehr-Rye et al. 2017); the PROXI study demonstrated a higher 2-year mortality in patients with abdominal malignancy who received an FIO_2_ of 0.8; and similarly, in 2018, a trial of over 5000 patients reported that using an FIO_2_ of 0.8 intraoperatively instead of 0.3 did not alter SSI rates but did double 30-day mortality rates (*p* = 0.08) (Kurz et al. 2018). Secondly, continuous monitoring of arterial oxygenation during general anaesthesia occurs mainly via pulse oximetry with a scale that stops at 100%. New technology may allow non-invasive measurement of surrogate markers of PaO_2_ in the future (e.g. the oxygen reserve index (Applegate et al. 2016)), but the use of these devices is currently limited. The duration of oxygen exposure may also possibly affect the outcomes, yet this has often not been considered or reported in clinical trials previously. The method of determining cumulative oxygen dose demonstrated here could represent a more relevant measure for use in future outcome studies.

### Strengths and limitations

This study characterises how anaesthetists in the UK currently use oxygen during a mixed selection of major surgery and in a large number of different hospitals. The biggest limitation to these findings is that corresponding clinical outcomes could not be collected. This should be a focus of future prospective research studies, and although this study was never designed to collect outcome data itself, our findings that FIO_2_ of 0.5 currently represents “standard care” (and not 0.3 as used by most trials to date) should be considered in the design of future trials. Area under the curve analysis could only be performed between times of arterial blood gas sampling, which could not be specified due to the retrospective and observational study design, and consequently, our data may represent underestimates of total cumulative oxygen doses as FIO_2_ is often increased at the start and end of anaesthesia to prepare for intubation and extubation. Because of the way most recruiting hospitals routinely document anaesthesia, the majority of data were collected from paper anaesthetic records. Previous studies have suggested a paper anaesthetic chart is not always the most accurate record of intraoperative events (Devitt et al. 1999); however, in our study, findings from centres using paper charts were still very similar to those using electronic recording systems. In order to record PaO_2_ values, we included patients undergoing procedures necessitating arterial line insertion; implying our findings might only be applicable to those in whom invasive monitoring was deemed necessary by the anaesthetist, either due to patient or operative factors. However, despite all of these limitations, our findings corroborated reports of current practice in other countries exactly (Kurz et al. 2018).

## Conclusions

Anaesthetists are currently faced with an international recommendation on the intraoperative administration of oxygen that conflicts with the majority of evidence from other clinical contexts. It is perhaps not surprising therefore that the amount of oxygen administered to patients undergoing general anaesthesia in the UK varies widely. The administration of an FIO_2_ of 0.5 appears to be the current standard of care for UK-based anaesthetists, which is often associated with moderate levels of hyperoxaemia intraoperatively. These findings are very similar to the reports from other countries and need to be considered in the design of any future studies investigating the potential impact intraoperative oxygen therapy may have on surgical patients’ outcomes.

## Abbreviations

ABG: Arterial blood gas
DGH: District general hospital
F_I_O_2_: Fractional inspired oxygen concentration
IQR: Interquartile range
PaO_2_: Arterial oxygen partial pressure
PEEP: Positive end-expiratory pressure
PLAN: Pan-London Perioperative Audit and Research Network
ROS: Reactive oxygen species
SPARC: South Coast Perioperative Audit and Research Collaborative
SpO_2_: Peripheral oxygen saturation
SSI: Surgical site infection
WHO: World Health Organization
