# Correction to: Intraoperative oxygenation in adult patients undergoing surgery (iOPS): a retrospective observational study across 29 UK hospitals

**DOI:** 10.1186/s13741-018-0106-7

**Published:** 2018-10-24

**Authors:** Clare M. Morkane, Helen McKenna, Andrew F. Cumpstey, Alex H. Oldman, Michael P. W. Grocott, Daniel S. Martin, Pan London Perioperative Audit and Research Network (PLAN), South Coast Perioperative Audit and Research Collaboration (SPARC)

**Affiliations:** 1 Division of Surgery and Interventional Science (University College London) and Royal Free Perioperative Research Group, Department of Anaesthesia, Royal Free Hospital, 3rd Floor, Pond Street, London, NW3 2QG UK; 2 University of Southampton/University Hospital Southampton and NIHR Biomedical Research Centre, Tremona Rd, Southampton, SO16 6YD UK; 3 University Hospital Southampton, Tremona Rd, Southampton, SO16 6YD UK

## Correction

Following publication of the original article (Morkane et al., 2018), it was noticed that the second collaborating group ‘South Coast Perioperative Audit and Research Collaboration (SPARC)’ was not displayed on the HTML online version due to an error by the Publisher. This collaborating group however does display on the PDF of the original article.

The correct author list is as follows:

Clare M. Morkane^1†^, Helen McKenna^1†^, Andrew F. Cumpstey^2†^, Alex H. Oldman^3^, Michael P. W. Grocott^2^, Daniel S. Martin^1*^ and Pan London Perioperative Audit and Research Network (PLAN), South Coast Perioperative Audit and Research Collaboration (SPARC).
